# Supplementary figures and images for: Oversized cells activate global proteasome-mediated protein degradation to maintain cell size homeostasis
Source: eLife. 2025 Jan 10;14:e75393. doi: 10.7554/eLife.75393 (PMC11810107; doi:10.7554/eLife.75393)

## Slide 1
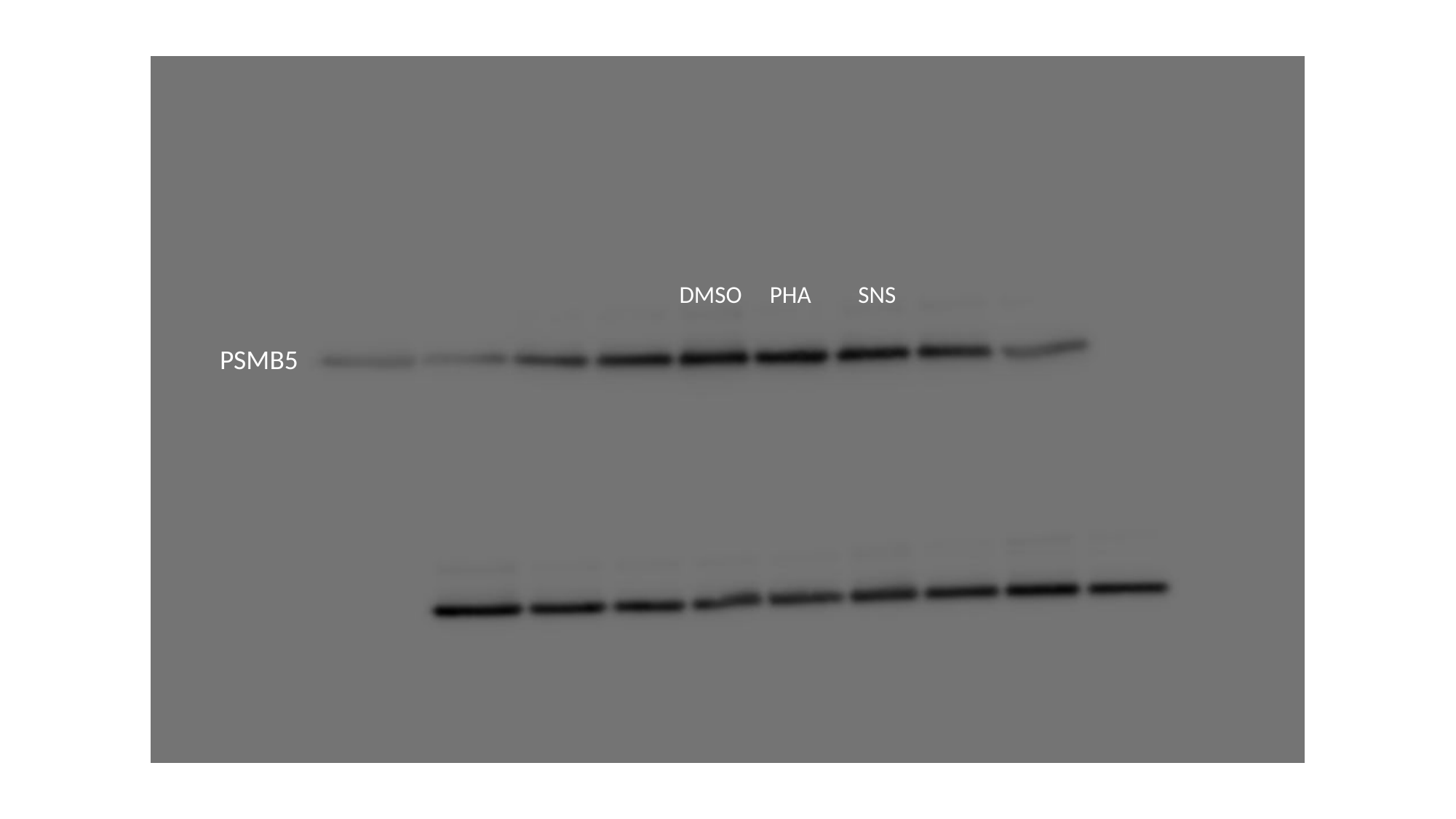

DMSO
PHA
SNS
PSMB5

## Slide 2
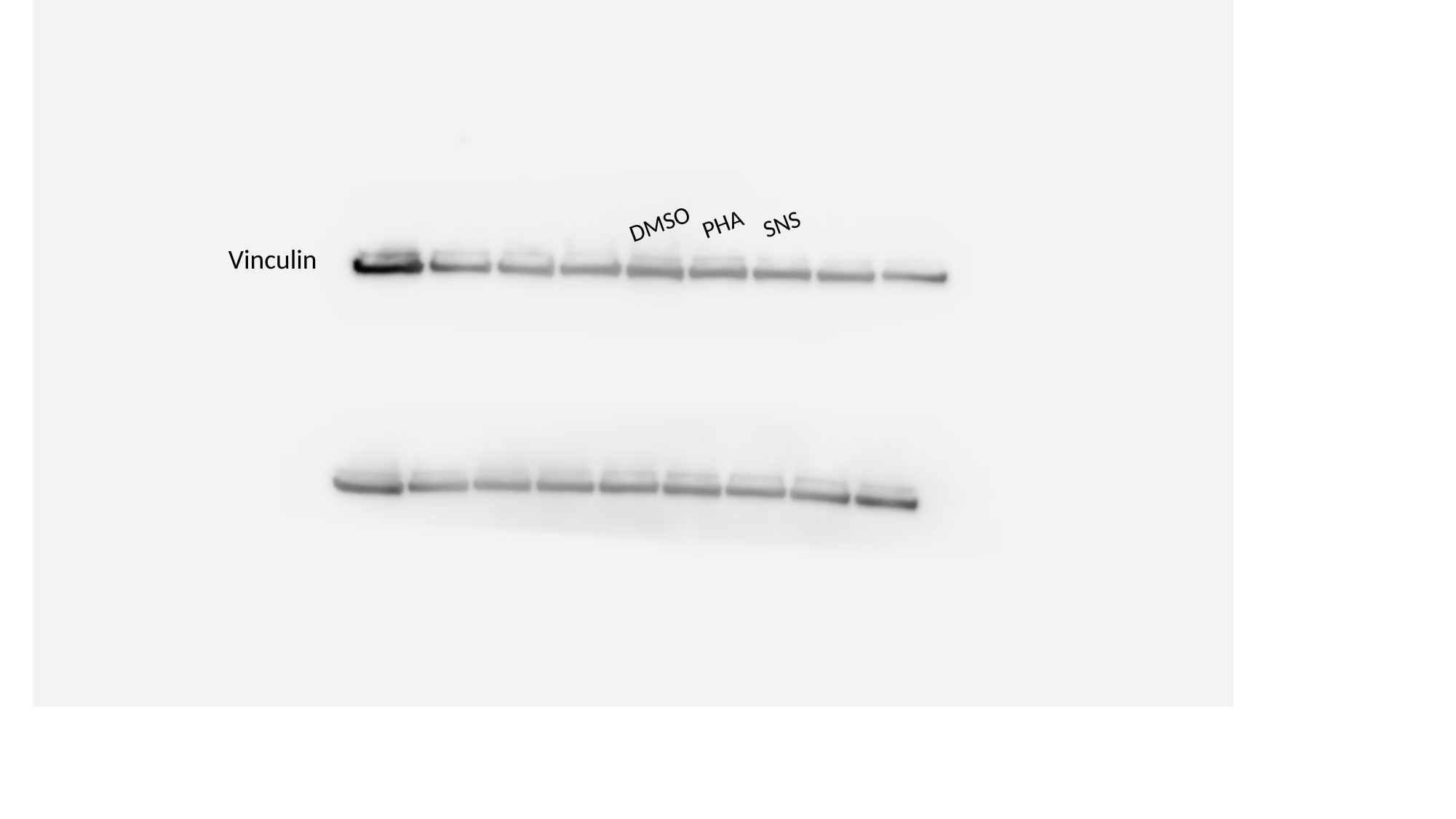

SNS
DMSO
PHA
Vinculin

Supplement: Figure 3—figure supplement 5—source data 1. [file elife-75393-fig3-figsupp5-data1.zip › Figure 3_5/Figure3S5A_Data_Labeled.pptx]

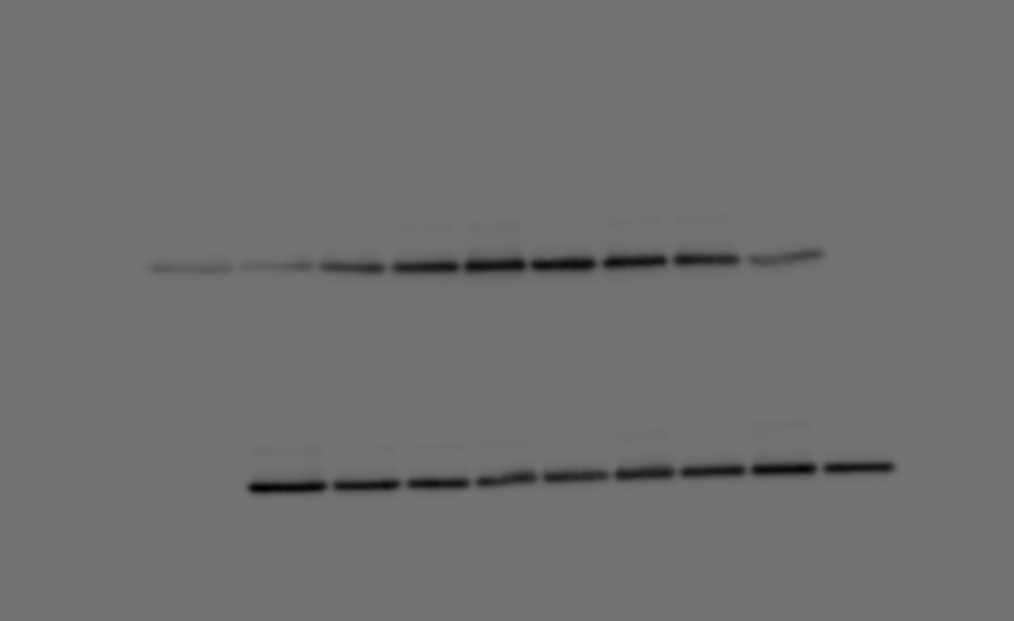

Supplement: Figure 3—figure supplement 5—source data 2. [file elife-75393-fig3-figsupp5-data2.zip › Figure 3_6/Figure3S5A_Data_PSMB5_TopRowLanes5to7.tif]

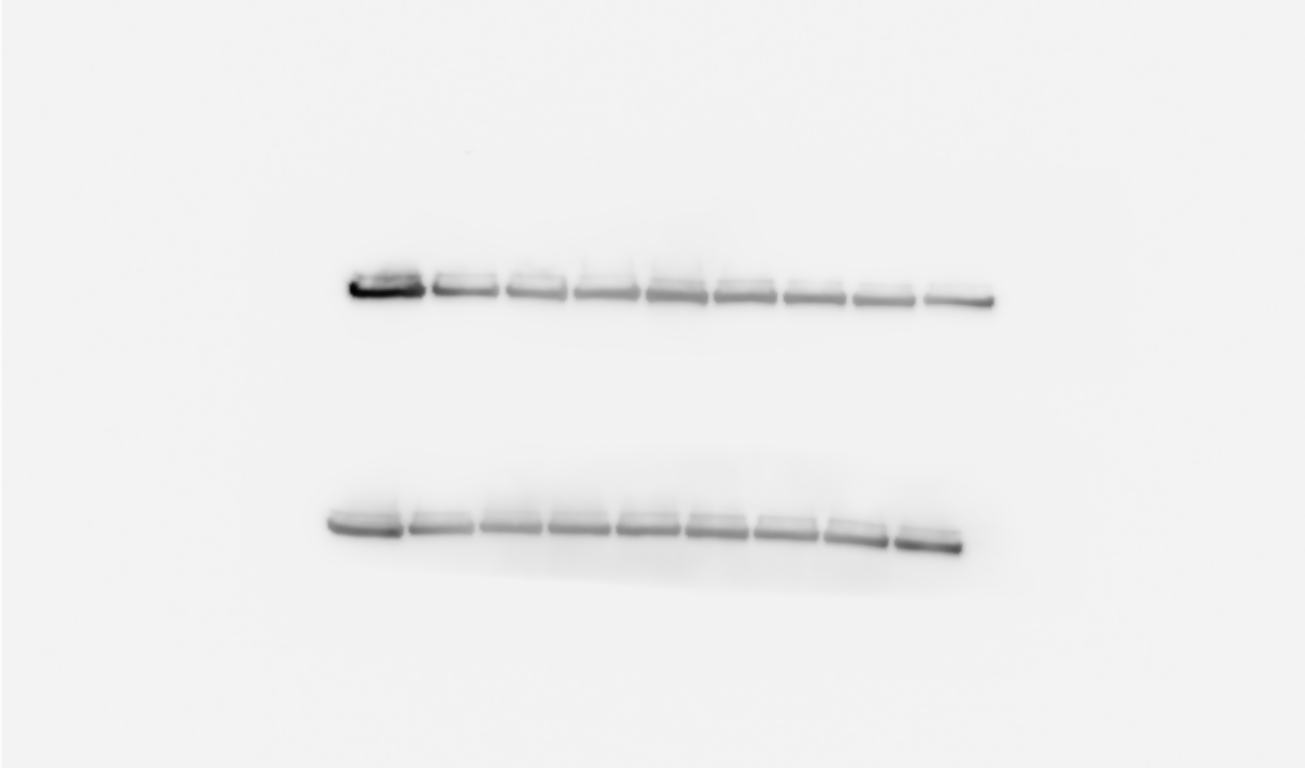

Supplement: Figure 3—figure supplement 5—source data 2. [file elife-75393-fig3-figsupp5-data2.zip › Figure 3_6/Figure3S5A_Data_Vinculin_TopRowLanes5to7.tif]
